# Female zebra finches prefer the songs of males who quickly solve a novel foraging task to the songs of males unable to solve the task

**DOI:** 10.1002/ece3.6690

**Published:** 2020-08-20

**Authors:** Clara Howell, Rindy Anderson, Elizabeth P. Derryberry

**Affiliations:** ^1^ Department of Ecology and Evolutionary Biology Tulane University New Orleans LA USA; ^2^ Department of Ecology and Evolutionary Biology University of Tennessee Knoxville TN USA; ^3^ Department of Biological Sciences Florida Atlantic University Davie FL USA

**Keywords:** cognition, foraging task, mate choice, sexual selection, *Taeniopygia guttata*, zebra finch

## Abstract

Correlative evidence suggests that high problem‐solving and foraging abilities in a mate are associated with direct fitness advantages, so it would benefit females to prefer problem‐solving males. Recent work has also shown that females of several bird species who directly observe males prefer those that can solve a novel foraging task over those that cannot. In addition to or instead of direct observation of cognitive skills, many species utilize assessment signals when choosing a mate. Here, we test whether females can select a problem‐solving male over a non‐solving male when presented only with a signal known to be used in mate assessment: song. Using an operant conditioning assay, we compared female zebra finch (*Taeniopygia guttata*) preference for the songs of males that could quickly solve a novel foraging task to the songs of males that could not solve the task. Females were never housed with the test subject males whose song they heard, and the only information provided about the males was their song. We found that females elicited more songs of problem‐solving males than of non‐solvers, indicating that song may contain information about a male’s ability to solve a novel foraging task and that naïve females prefer the songs of problem‐solving males.

## Graphical abstract

Birdsong is known to be an indicator of overall brain function and developmental conditions, but it is unknown whether song can reflect cognitive or personality traits that specifically affect foraging tasks. Here, we tested female preference for song from males who could quickly solve or could not solve an artificial novel foraging task. Although females had no prior knowledge of males, they solicited more song from males who could quickly solve the novel foraging task.

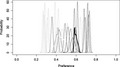

## 1 INTRODUCTION

A key question in the study of animal cognition is whether sexual selection has contributed to its evolution (Boogert, Fawcett, Fawcett, & Lefebvre, 2011; Darwin, 1871). A related but subtly different question is whether signals used in mate choice reflect cognitive abilities that are under natural selection, and would therefore offer direct or indirect benefits in a potential mate. The extension of this question is whether the utility of certain assessment signals in selecting a mate with beneficial cognitive abilities has led to their continued use and evolution.

The hypothesis that signals used in mate choice (“assessment signals”) serve as indicators of male quality has been extensively supported in a wide variety of taxa (reviewed in Wilgers & Hebets, 2015), including fish (Houde & Torio, 1992; Karino, Utagawa, & Shinjo, 2005), birds (Doucet & Montgomerie, 2003; Hill, 1991), insects (David, Bjorksten, Fowler, & Pomiankowski, 2000; Holzer, Jacot, & Brinkhof, 2003), and even humans (Hume & Montgomerie, 2001; Little, Jones, DeBruine, & Feinberg, 2008). The hypothesis that assessment signals can serve as an indicator of general brain function has also been supported (Boogert, Fawcett, et al., 2011), in particular by studying the effects of developmental stress on bird song (Peters, Searcy, & Nowicki, 2014). Developmental stress lowers cognitive performance in a range of taxa (Erhard, Boissy, Rae, & Rhind, 2004; Kitaysky, Kitaiskaia, Piatt, & Wingfield, 2006; Kriengwatana, MacDougall‐Shackleton, Farrell, Garcia, & Aitken, 2015; Levitsky & Strupp, 1995; Peters et al., 2014; Santos de Souza et al., 2008) and, specifically within birds, on a range of tasks including novel foraging tasks (Kitaysky et al., 2006), spatial learning tasks (Kriengwatana et al., 2015; Pravosudov, Lavenex, & Omanska, 2005), associative learning tasks (Farrell, Morgan, & MacDougall‐Shackleton, 2016; Fisher, Nager, & Monaghan, 2006), and auditory learning tasks (Farrell et al., 2016). Developmental stress also negatively impacts preferred features of song, such as complexity (Buchanan, Leitner, Spencer, Goldsmith, & Catchpole, 2004; Spencer, Buchanan, Goldsmith, & Catchpole, 2003), bout duration (Buchanan, Spencer, Goldsmith, & Catchpole, 2003), and accuracy in relation to tutor song (Nowicki, Searcy, & Peters, 2002), and is less preferred by females when presented in comparison with song from developmentally robust males (Searcy, Peters, Kipper, & Nowicki, 2010; Spencer et al., 2005).

These findings suggest that song can serve as an indicator of gross brain quality and function in the case of developmental inequity between potential mates. However, in the absence of conditions like developmental stress or genetic abnormalities that cause widespread disruption in the body and brain, there is increasing evidence that avian intelligence is more modular than general in nature (Searcy & Nowicki, 2019). For instance, there are seldom correlations between performance on different cognitive tasks within individuals (Anderson et al., 2017; DuBois, Nowicki, Peters, Rivera‐Cáceres, & Searcy, 2018; van Horik & Madden, 2016; MacKinlay & Shaw, 2018; Medina‐García, Jawor, & Wright, 2017; Nettle et al., 2015), with some exceptions (Ashton, Ridley, Edwards, & Thornton, 2018; Shaw, Boogert, Clayton, & Burns, 2015). Notably, exceptions tend to occur in wild populations where unequal developmental conditions are more likely. Because of the apparent modularity of avian intelligence, it is unclear whether song is a reliable indicator of the specific cognitive skills that would offer females fitness benefits if found in a potential mate.

One such cognitive skill is foraging ability. Foraging efficiency has been shown to increase fitness in a range of avian species (Cole, Morand‐Ferron, Hinks, & Quinn, 2012; Lemon & Barth, 1992; Lescroël et al., 2010; Orians, 1969; Shaw, MacKinlay, Clayton, & Burns, 2019; Weathers & Sullivan, 1989) and is a particularly important skill for a mate in monogamous species that engage in biparental food provisioning. Foraging ability has also been shown to affect mate preferences in crossbills (*Loxia curvirostra*), budgerigars (*Melopsittacus undulatus*), and zebra finches (*Taeniopygia guttata*) when females directly observe males solving a foraging task (Chantal, Gibelli, & Dubois, 2016; Chen, Zou, Sun, & ten Cate, 2019) or more efficiently extracting food from a source (Chantal et al., 2016; Snowberg & Benkman, 2009). Foraging ability is also correlated with problem‐solving on artificial tasks in the wild (Cauchard, Boogert, Lefebvre, Dubois, & Doligez, 2013; Cole et al., 2012), which has in turn been correlated with reproductive success (Ashton et al., 2018; Cauchard et al., 2013; Cole et al., 2012; Keagy, Savard, & Borgia, 2009).

The ability to solve a novel foraging problem is thus related to reproductive success and has been shown in some species to be preferred by females when observed directly. However, it remains unclear whether the same signals that can indicate gross brain function, as determined by developmental conditions, can reflect this specific skill. Do songs contain information about male foraging ability, given the apparent modularity of avian cognition, and could this help explain the utility of song as an assessment signal? Or, conversely, does song only contain information about gross developmental conditions, and not more specific skills that would offer females direct fitness benefits?

To answer these questions, we tested whether female zebra finches would discriminate between males with superior versus inferior performance on a novel foraging task based solely on their song. Using an operant conditioning assay, we compared female preference for the songs of males that quickly solved the novel foraging task to the songs of males that were incapable of solving the task. The females had no exposure to the males whose songs they were tested with, thus isolating their knowledge of the males to information contained in their songs. We predicted that if song is a reliable signal of novel foraging skill, females would be able to identify and prefer songs from males who were capable of quickly solving a novel foraging task over songs from males who were unable to solve the task.

## 2 METHODS

### 2.1 Subjects and housing conditions

To assay male foraging ability and acquire recordings of male songs, we obtained 25 adult male zebra finches of unspecified age from Magnolia Farms avian breeder (Anaheim, CA). Birds were housed in a group cage for four days upon arrival, then weighed with a Pesola scale and placed in individual cages in a group room. Cages were wire and measured 48 cm × 25 cm × 30 cm with two perches and one cuttlebone each. Food and water were provided ad libitum. Vivarium rooms were illuminated on a 13:11 light–dark cycle. Temperature in the room was maintained at 22°C. Housing conditions were approved by Tulane IACUC Protocol 0427R.

To measure female preference, we obtained 15 adult female zebra finches of unspecified age, also from Magnolia Farms. Females were purchased 18 months after the male zebra finches were purchased to minimize the possibility of prior interaction between males and females due to overlap at Magnolia Farms. Housing conditions were identical to the first group of birds. Males were not housed in the facility at the same time as the females, and females were not exposed to their songs prior to testing.

### 2.2 Novel foraging assay

To solve the novel foraging task, males learned to remove lids covering baited wells in a block. The block was 10 × 14 cm and composed of gray composite plastic with six drilled wells (1.7 cm diameter × 1 cm depth). The food reward consisted of 2–3 millet seeds placed in the bottom of 4 of 6 wells. Baiting patterns were randomly generated with R (R Development Core Team, 2016) and changed between each trial. Lids were made of blue and yellow round plastic counting chips glued to a round rubber bottom, such that lids fitted into the wells and needed to be lifted off in order for the bird to obtain the food reward. Stages consisted of: (1) just the block, (2) lids placed adjacent to, but not covering baited wells, (3) lids half‐covering wells, (4) lids tipped into wells, and (5) lids completely covering and fitted into wells. Testing was done in the housing room and birds remained in auditory contact with their flockmates to alleviate stress caused by moving or social isolation. Dividers were placed between cages during testing so that neighbors of the study subject could not see the task beforehand.

Trials consisted of two‐minute periods in which the block was placed in the cage and the bird was allowed to interact with it. After each two‐minute trial, the block was removed from the cage for approximately 10 min while other birds were tested. Each bird was given six trials per day. Food was removed 5 hr before testing to increase motivation. After each round of testing, a motivation check was done in which a food dish was placed in the cage and the time it took the bird to approach and eat from it was recorded. If birds ate from food dishes in less than one minute, they were considered sufficiently motivated. All birds passed all motivation tests, indicating that food removal left them sufficiently motivated to obtain a food reward. Neophobia was measured as either the latency (number of trials) it took to pass the first stage, in which the block was presented for the first time, or the second stage, in which lids were presented for the first time.

Birds had to eat from at least two baited wells to pass a trial and had to pass three out of four consecutive trials to move on to the next stage. Passing criteria remained the same throughout the novel foraging task. If birds were stuck on a particular stage for more than four days, they were moved back to a previous stage or, for stage 1 only, the block was left in the cage overnight. If a bird did not complete a stage (not the entire task, just one stage) within 60 trials, it was removed from trials and designated a Non‐Solver, as further food removal and testing were potentially harmful to birds and in our judgment, these individuals were not likely to progress further.

### 2.3 Stimulus sets, song recording, and song complexity analysis

The six best‐performing males (those that learned the task in the fewest number of trials) were designated Solvers, while the six worst‐performing males (those that did not solve the task) were designated Non‐Solvers. Six stimulus sets were created by randomly pairing 1 Solver song with 1 Non‐Solver song. All Solvers used as stimulus males solved the task in under 30 trials (mean = 22.67 trials); all Non‐Solvers took at least 60 trials before they were pulled from testing (mean = 90.67 trials). The six fastest Solvers were chosen to maximize disparity between the abilities of Solvers and Non‐Solvers. Five out of the six Non‐Solvers dropped out during stage 5, in which they could no longer see the food reward. The remaining Non‐Solver dropped out during stage 1, in which food was placed in wells. Because of concern that this Non‐Solver was categorically different in ability than the five Non‐Solvers who failed at the same stage, statistics were run on female preference including and not including this stimulus set. Stimulus Solver and Non‐Solver males were recorded in sound attenuation chambers (Industrial Acoustics) using Shure SM57 directional microphones and Sound Analysis Pro (see Tchernichovski, Nottebohm, Ho, Pesaran, & Mitra, 2000). Males were placed in divided cages with a female (not a study subject and not housed with study subjects) in the other half of the cage in order to elicit directed song (ten Cate, 1985). Males and females were thus in visual and auditory contact but not physical contact. To acclimate, pairs were placed in sound attenuation chambers for 24 hr prior to recording. Song output was recorded during the subsequent 24 hr. The majority of males produced hundreds of directed songs in this time period. Males that produced fewer songs were re‐recorded in separate sessions with different females until enough songs for analysis were produced. Female zebra finches have been shown to prefer longer songs (Neubauer, 1999), so we selected the longest song from each male's repertoire.

Zebra finch recordings were analyzed after the experiment using Raven Pro Interactive Sound Analysis Software (The Cornell Lab of Ornithology, version 1.5; Bioacoustics Research Program, 2014). Typically, song complexity is analyzed by selecting random motifs from across a male's repertoire and averaging their complexity values (e.g., Boogert, Giraldeau, & Lefebvre, 2008). However, we were not interested in whether problem‐solving ability correlated with general song complexity in males, but in whether song complexity explained the female preference we observed. We thus analyzed only the songs used as stimuli. We measured several potential measures of complexity in this species: song length, number of phrases, average phrase length, average elements per phrase, and total unique elements per song. Data were collected by visual inspection of spectrograms (256 pt. transform, frequency resolution = 86.1 Hz), and elements were categorized following Airey and DeVoogd (2000). To categorize elements as same or different, we used characteristics of the number and distribution of harmonics, frequency modulation, and element duration. Introductory elements were counted as phrases but excluded from phrase length and elements per phrase analyses.

### 2.4 Female choice assay

Prior to the mate choice assay, each female's cage was moved into a sound attenuation chamber equipped with one audio speaker. Females were given 24 hr to habituate to the chambers, at which point operant perches were installed on either side of the front cage door, so that total perches in the cage included two operant perches and two normal perches placed diagonally in the back of the cage. Operant perches were placed approximately 10 cm above the cage floor and 25 cm apart. Perches were approximately 9 cm long, ½ cm in diameter, and made of wood. Perches were attached to Honeywell lever arm depression micro‐switches. The perch on the left when facing the cage was designated perch A, and the perch on the right was designated perch B. Hopping on a perch caused the perch to lower slightly and trigger a pressure‐sensitive button on the micro‐switch, which in turn triggered playback of a song through the speaker. Each hop on the perch triggered playback of one song; if the bird remained on the perch no additional songs played and if birds left the perch, the song continued in its entirety. In this type of operant conditioning, the song itself serves as the reward and encourages females to continue eliciting song through hops (Anderson, 2009; Riebel, 2000; Stevenson, 1967). All songs were volume‐adjusted to 65 dB SPL at the chamber center. All operant data were collected through Sound Analysis Pro (see Tchernichovski et al., 2000).

Females learned to trigger song during an initial training period (Anderson, Peters, & Nowicki, 2014). For training, females were presented with conspecific/heterospecific song pairs (heterospecific song was from rufous‐collared sparrows, *Zonotrichia capensis*). Whether perch A played conspecific and perch B played heterospecific, or vice versa, was alternated between birds, and switched every day for the same bird. Switching songs between perches helped control for side bias. In order to pass training, females had to hop on both perches for two days in a row. All females passed training within 4 days.

After training, females were presented with Solver/Non‐Solver stimuli. Females were given a new pair of Solver and Non‐Solver songs every day, in which a single song from a Solver male was triggered by a hop on one perch, and a single song from a Non‐Solver male was triggered by a hop on another perch. The side that played Solver song was switched each day to control for side bias. Each bird heard 3 or 4 of 6 Solver/Non‐Solver pairs, as female response tends to drop off after prolonged periods in preference testing. Testing multiple stimulus sets on each female also greatly reduces the risk that a chosen stimulus set is non‐representative of its category and yet repeatedly measured (i.e., pseudoreplication in acoustic experiments; Kroodsma, 1989). Trials ran from 11 a.m. to 5 p.m. each day, during which the number of hops on each perch was recorded. Outside of trial times, hops on a perch did not trigger playback.

### 2.5 Statistical analysis

Preference data are often analyzed using non‐parametric analyses, such as the Wilcoxon sign‐rank test, in which preferences are expressed as ratios and compared to a null. However, this approach has several drawbacks. For example, (a) it does not measure the strength of preferences, only whether they are statistically different from the null, (b) it fails to account for different counts per replicate and does not adjust confidence intervals accordingly, and (c) it does not model variation in individual‐level choice—for instance, if half of individuals in a population strongly prefer one option and half another, the population preference will still be 0.5 and may not differ significantly from the null (Fordyce, Gompert, Forister, & Nice, 2011). Given these drawbacks, we chose to use a hierarchical Bayesian model designed for ecological preference and count data, implemented using the R packages bayespref (version 1.0) and coda (version 1.0; Fordyce et al., 2011; Plummer, Best, Cowles, & Vines, 2006). The benefit of the hierarchical Bayesian model is that it directly estimates the strength of preference, appropriately models uncertainty, and gives estimates of both individual‐ and population‐level preferences.

The R package bayespref uses Markov chain Monte Carlo (MCMC) to first assess the probability of the experimental count data given an individual preference, thus obtaining a model of individual preference, and at each step in the chain further assesses the probability of an individual preference given a population preference, thus obtaining a model of population preference. In a two‐choice scenario like ours, individual preferences are modeled as binomial distributions and population preferences as beta distributions. The first model was run using bayespref for female preference for conspecific versus heterospecific song, to confirm that females were showing the expected preference for conspecific song and that the operant trials were reflecting preference. The second model was run for Solver versus Non‐Solver song to determine whether females showed a population‐level preference for Solver song. Models were run with 10,000 generation MCMC chains with 1,000‐generation burn‐ins. Mixing of search chains was verified via diagnostic plots of MCMC samples and checking the effective sample size.

In the first model, we used data for each female, where the total number of conspecific hops over two days was modeled as one possible outcome and the total number of heterospecific hops over two days was modeled as the alternative outcome. In the second model, we also had data for each female, where the total number of Solver hops over all test days was modeled as one possible outcome and the total number of Non‐Solver hops over all test days was modeled as the alternative outcome. This controlled for side bias, which in some females was noticeable even in conspecific versus heterospecific trials. To evaluate the impact of stimulus set on female preference, and to ensure that no one stimulus set was disproportionately affecting results, we ran a third hierarchical Bayesian model using stimulus song at the individual level and preference for all stimulus songs in a given category (Solver or Non‐Solver) at the population level. In this model, we had data for each stimulus set (1–6), where the total number of female hops across all trials by all birds for the Solver song was modeled as one possible outcome and the total number of female hops across all trials for the Non‐Solver song was modeled as the alternative outcome. In the same way that the first model calculated individual female preference and confirmed that no one female was driving population‐level preference, this model confirmed that no one song set was disproportionately “attractive” and driving preference for Solver song.

Because of concern that one of the Non‐Solvers was categorically different in ability than the other 5, we also ran these Bayesian models using only data from the 5 stimulus sets in which the Non‐Solvers all failed at the same stage.

Although we considered the Bayesian approach sufficient to answer our research questions, we also ran a mixed effects linear model using the preference data for each trial and including stimulus ID, female ID, and order of presentation as random effects. The results of this model can be found in Appendix S4. This approach also found a significant difference between preferences for Solvers and Non‐Solvers and made no qualitative difference to our interpretation of the results.

We further tested whether any measures of song complexity (song length, number of phrases, phrase length, number of elements, or number of unique elements) differed between stimulus pairs. To test this, we used paired *t* tests. We corrected for multiple testing (here, five song variables) using a Bonferroni correction, such that the adjusted alpha was 0.01.

Because we were interested in whether neophobia affected performance, we also tested whether Non‐Solvers took longer than Solvers to solve the first stage of the task in which they were introduced to the block, and the second stage in which they were introduced to the lids, using an independent *t* test. All statistical analyses were performed in R (Version 3.5.0; R Development Core Team, 2016).

## 3 RESULTS

### 3.1 Novel foraging task results

25 males were assessed with the novel foraging assay, of which 7 were unable to complete the task within the established cutoff (birds that did not pass any given stage within 60 trials were pulled from testing). Of those males that did pass, it took an average of 43.61 trials (*SD* = 22.40 trials) to do so. Solvers used for stimuli were selected from the 6 fastest males to solve the task, and took an average of 22.67 trials to solve the task. Six out of seven non‐solvers were used for stimuli, and all Non‐Solvers took at least 60 trials before they were pulled from testing (average = 90.67 trials). Full results from the novel foraging task can be found in Appendix S1.

### 3.2 Females preferred conspecific song to heterospecific song

**FIGURE 1:**
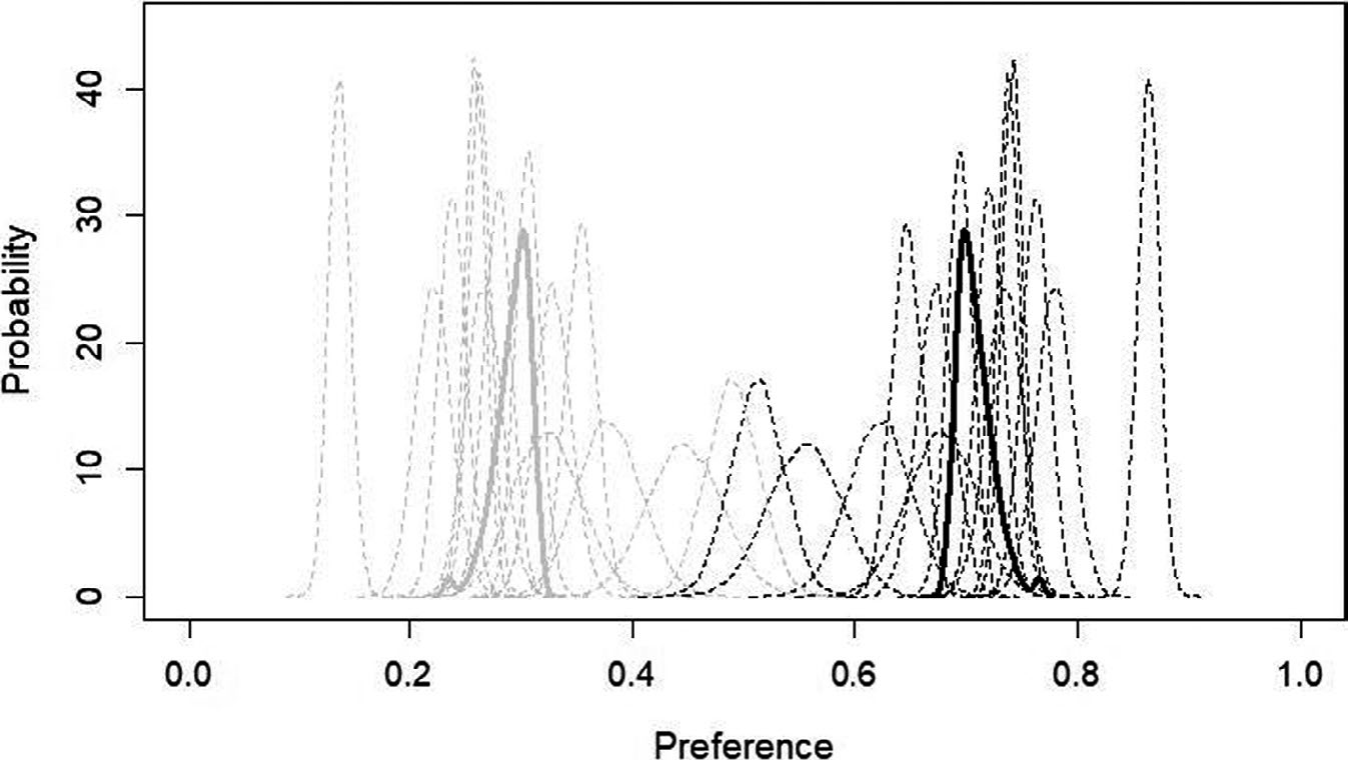
Probability densities of conspecific preference estimates from hierarchical Bayesian statistical model for conspecific (black) versus heterospecific (light gray) song. Solid lines indicate population‐level estimates for conspecific song preference; dotted lines indicate individual‐level estimates for conspecific song preference. Preference of 1.0 indicates 100% preference, which would mean that the bird hopped exclusively on perches triggering the given stimulus type. Preference of 0.0 indicates 0% preference, which would mean that the bird did not hop at all on the perches triggering the given stimulus type. Population preference for conspecific song was 0.705 (95% credible intervals: 0.687, 0.748) and population preference for heterospecific song was 0.295 (95% credible intervals: 0.252, 0.313). Model was run for 10,000 generations with a 1,000 generation burn‐in

The first Bayesian model (Figure 1) confirmed that females show a preference for conspecific song in the operant chambers. Population preference for conspecific song was 0.705 (95% credible intervals: 0.687, 0.748) and population preference for heterospecific song was 0.295 (95% credible intervals: 0.252, 0.313). Effective sample size (ESS), a measure of the MCMC function and not associated with sample size in a typical non‐parametric analysis, was 1,593.247. Average hops per conspecific/heterospecific trial was 521.93 (*SD* = 300.96). Full results from the conspecific/heterospecific preference trials can be found in Appendix S3.

### 3.3 Females preferred songs produced by males that solved the novel foraging task

**FIGURE 2:**
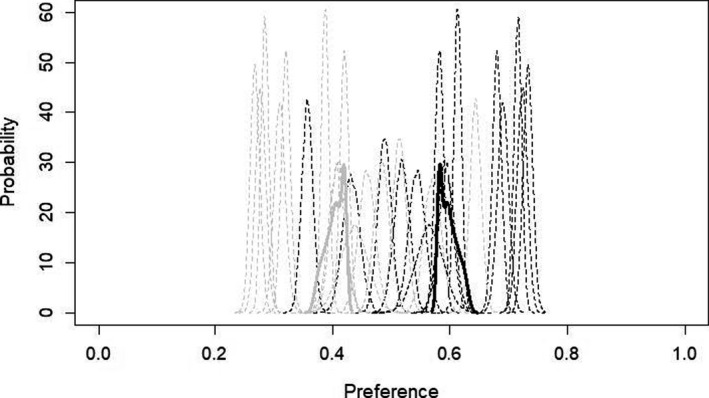
Probability densities of Solver preference estimates from hierarchical Bayesian statistical model for Solver (black) versus Non‐Solver (light gray) song, where song preferences were aggregated for each female. Solid lines indicate population‐level estimates for Solver song preference; dotted lines indicate individual‐level estimates for Solver song preference. Preference of 1.0 indicates 100% preference, which would mean that the bird hopped exclusively on perches triggering the given stimulus type. Preference of 0.0 indicates 0% preference, which would mean that the bird did not hop at all on the perches triggering the given stimulus type. Population preference for Solver song was 0.594 (95% credible intervals: 0.581, 0.628), and population preference for Non‐Solver song was 0.406 (95% credible interval: 0.372, 0.419). Model was run for 10,000 generations with a 1,000 generation burn‐in

The second Bayesian model (Figure 2) showed that females preferred Solver song and gave information about strength of preference. Population preference for Solver song was 0.594 (95% credible intervals: 0.581, 0.628), and population preference for Non‐Solver song was 0.406 (95% credible interval: 0.372, 0.419). ESS was 1,674.475. In a Bayesian preference model, confidence that one group is preferred over another increases as overlap in the credible intervals of both groups declines. In our model, there was no overlap between the 95% credible intervals for Solver preference and Non‐Solver preference, indicating a substantially higher preference for the song of Solver males, with a 5% or lower probability that Non‐Solver song was preferred equally or more than Solver song (Fordyce et al., 2011). For comparison, 95% credible intervals for group conspecific song preference were 0.687, 0.748; and 95% credible intervals for group heterospecific song preference were 0.252, 0.313 (see Figure 1). There was a greater difference between conspecific and heterospecific credible intervals, but preference for conspecific song was only approximately 11 percentage points higher (0.705) than preference for Solver song (0.594).

These patterns remained consistent when the 6th stimulus set was dropped (Solver preference: 0.581, 95% credible interval: 0.567, 0.617; Non‐Solver preference: 0.419, 95% credible interval: 0.383, 0.433).

In Solver/Non‐Solver trials, the average number of hops per trial was 704.84 (*SD* = 384.59). Full results from Solver/Non‐Solver trials can be found in Appendix S3.

### 3.4 Females preferred the Solver song in all stimulus sets

**FIGURE 3:**
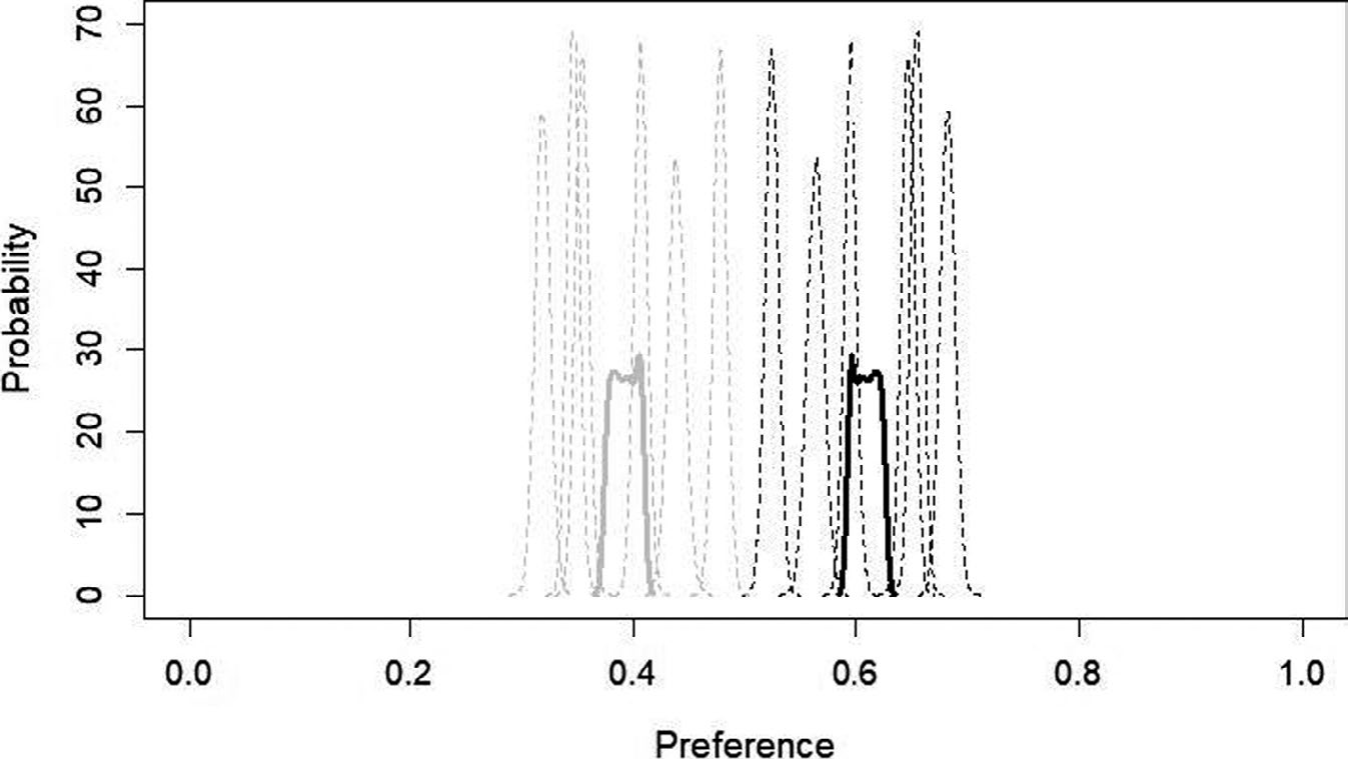
Probability densities of Solver song preference estimates from hierarchical Bayesian statistical model for Solver (black) versus Non‐Solver (light gray) song, where female preferences were aggregated for each song. Solid lines indicate group (Solver) level estimates; dotted lines indicate individual (stimulus song) level estimates. Preference of 1.0 indicates 100% preference, which would mean that the bird hopped exclusively on perches triggering the given stimulus type. Preference of 0.0 indicates 0% preference, which would mean that the bird did not hop at all on the perches triggering the given stimulus type. Aggregate preference for Solver song was 0.608 (95% confidence intervals: 0.592, 0.625). Aggregate preference for Non‐Solver song was 0.392 (95% confidence intervals: 0.374, 0.408). Model was run for 10,000 generations with a 1,000 generation burn‐in

The third Bayesian model (Figure 3) confirmed a general preference for Solver songs and showed that in each stimulus set, Solver song was preferred over Non‐Solver song. Aggregate preference for Solver song was 0.608 (95% credible intervals: 0.592, 0.625). Aggregate preference for Non‐Solver song was 0.392 (95% credible intervals: 0.374, 0.408). In the stimulus set that was furthest apart in preference, Solver song had an estimated preference of 0.682 (95% credible intervals: 0.669, 0.695) while Non‐Solver song had an estimated preference of 0.318 (95% credible intervals: 0.305, 0.331). In the stimulus set that was closest in preference, Solver song had an estimated preference of 0.523 (95% credible intervals: 0.551, 0.535), while Non‐Solver song had an estimated preference of 0.477 (95% credible intervals: 0.465, 0.489). Even in the closest stimulus set, 95% credible intervals did not overlap (thus making it a 5% or less chance that the Non‐Solver song was preferred over the Solver song). We can therefore say that Solver song was preferred in all stimulus pairs and that population preference for Solver song (see Figure 2) was not driven by one or two particularly attractive Solver songs, but by a consistent pattern in preference across all six stimulus sets.

These patterns remained consistent when the 6th stimulus set was dropped (Solver preference by stimulus set: 0.597, 95% credible interval: 0.583, 0.613; Non‐Solver preference by stimulus set: 0.403, 95% credible interval: 0.387, 0.417).

### 3.5 Song complexity does not appear to explain female preference for Solver song

Solver and Non‐Solver songs did not differ significantly in any measures of complexity: song length (average Solver song length = 4.5 s, average Non‐Solver song length = 3.97 s; *T* = 0.384, *df* = 5, *p* = .461), number of phrases (average Solver phrases = 5.83, average Non‐Solver phrases = 4; *T* = 3.84, *df* = 5, *p* = .012), phrase length (average Solver phrase length = 0.834 s, average Non‐Solver phrase length = 0.844 s; *T* = −0.052, *df* = 5, *p* = .961), number of elements per phrase (average Solver elements = 5.25, average Non‐Solver elements = 4.92; *T* = 0.353, *df* = 5, *p* = .739), and number of unique elements (average Solver unique elements = 5.33, average Non‐Solver unique elements = 5.5; *T* = −0.143, *df* = 5, *p* = .892). However, our power to determine an effect of one standard deviation given an alpha of 0.01 is 0.194, so we cannot say with confidence that Solvers and Non‐Solvers in general do not have a difference in song complexity. Full results from the song complexity analysis can be found in Appendix S2.

### 3.6 Neophobia does not appear to explain faster learning by Solvers

Five of six Non‐Solvers ate consistently from the baited wells of a novel object (stage 1). The Non‐Solver who did not eat from the grid was given a maximum score of 60 in comparisons. Non‐Solvers did not significantly differ from Solvers in the number of trials required to pass stage 1 in a *t* test (*T* = −1.58, *df* = 5.1, *p* = .173). However, Non‐Solvers did tend to require more trials to pass stage 1 than Solvers (Solver mean = 6 trials, Non‐Solver mean = 19.5 trials). Solvers did not differ from Non‐Solvers in the number of trials required to pass stage 2, in which the second group of novel objects, colorful lids, was presented (*T* = −1.27, *df* = 4, *p* = .273). Furthermore, performance on stage 1 was not correlated with performance on stage 2 (*r* = −.336, *p* = .342), indicating that Non‐Solvers were not consistently hesitant in their approach. However, our power to determine an effect of 0.5 standard deviations given an alpha of 0.05 is 0.635, so we cannot fully exclude differences in neophobia between the two groups as a contributor to female preference.

## 4 DISCUSSION

In our operant assay, females elicited the songs of males that quickly completed a novel foraging task more frequently than they elicited the songs of males unable to complete the task, indicating that the females preferred the songs of Solvers over the songs of Non‐Solvers. Past work has shown a strong correlation between song preferences measured via operant conditioning and copulation solicitation displays (Anderson, 2009), the latter being considered a fairly close proxy for mating preference (Holveck & Riebel, 2007). Thus our findings support the hypothesis that females prefer the assessment signal of a male better at a novel foraging task, and replicate patterns of preference found when females directly observe males completing a foraging task (Chantal et al., 2016; Chen et al., 2019; Snowberg & Benkman, 2009).

Our results suggest that if females chose to mate with the males whose song they preferred, they would be selecting Solver males more often than Non‐Solver males. However, the mechanism linking problem‐solving and song quality is still unknown. Although song was recorded after the novel foraging task, we think it highly unlikely that performing the task affected song, particularly as zebra finches have a single song that is crystallized in adolescence (Zann, 1996). Rather, the more plausible interpretation of our data is that a third factor is correlated with both song quality and performance on the novel foraging task, such as a specific cognitive ability or personality trait. We are unable to fully determine this factor as the causes of successful learning on artificial problem‐solving tasks remain murky and debated (Rowe & Healy, 2014). Performance on artificial novel foraging assays is thought to measure cognitive ability, but can also be affected by personality factors such as neophobia, boldness and individual differences in motivation (reviewed in Griffin, Guillette, & Healy, 2015). We assured that all males were food‐motivated by depriving them of food before testing and also confirmed that all males were sufficiently motivated to eat by measuring latency to feed once food was returned to the home cage, and thus we do not think that differences in food motivation can explain our results. We also examined one measure of neophobia, latency to approach a novel object (Bouchard, Goodyer, & Lefebvre, 2007; Cauchard et al., 2013; Shaw et al., 2015). We found no statistically significant differences in latency to approach a novel object between Solvers and Non‐Solvers and also did not find a correlation between latency to approach one novel object (the block) and another novel object (the colored lids), indicating that Non‐Solvers were not consistently slow to approach the task. Given the power of our analysis, we cannot definitively say that Solvers were less neophobic. However, we did not find evidence of neophobia, which is consistent with a recent meta‐analysis of personality and cognition in birds that found no correlation between fear of novel objects and performance on novel foraging assays across multiple species (Dougherty & Guillette, 2018). Thus, while we cannot rule out the possibility that motivation or neophobia affected performance on the task and that females were responding to differences in personality that were reflected in song, we suggest that the more likely interpretation of our data is that the novel foraging task measured some aspect of cognitive ability that was also reflected in song.

While females were evidently responding to differences in Solver versus Non‐Solver song, how that information is encoded in song remains unknown. Past research has examined whether features of song, such as complexity (Boogert et al., 2008; Templeton, Laland, & Boogert, 2014), repertoire size (Boogert, Anderson, Anderson, Peters, Searcy, & Nowicki, 2011; MacKinlay & Shaw, 2018; Sewall, Soha, Peters, & Nowicki, 2013), and species typicality (DuBois et al., 2018) correlate with cognitive performance. The majority of these (Boogert, Anderson, et al., 2011; DuBois et al., 2018; MacKinlay & Shaw, 2018; Templeton et al., 2014) found predominately null relationships between song macrofeatures and cognitive performance, and two (Boogert, Anderson, et al., 2011; Sewall et al., 2013) even found a significant inverse relationship. We measured the complexity of stimulus songs and found no statistically significant differences in song length, number of phrases, phrase length, number of elements, or number of unique elements between the stimulus songs produced by Solver and Non‐Solver males. Given the power of our analysis, we cannot say that problem‐solving ability is unrelated to song complexity in zebra finches in general, only that there was no significant relationship within our sample of stimulus songs. The finding that females significantly preferred Solver songs despite no apparent difference in song complexity suggests that females attended to some feature in the songs that we as researchers could or did not measure, as has been found before in assays of local versus foreign song (Anderson et al., 2014). Differences between the songs sung by the two groups of males could include variations in fine acoustic structure, such as minute changes in relative amplitude of harmonics and periods between amplitude peaks within an element, which have been shown to encode biologically relevant information in zebra finch calls (Prior, Smith, Lawson, Ball, & Dooling, 2018). This study further highlights the need for future studies of female preference to measure female response to stimuli in addition to quantifying and categorizing stimuli attributes, as perhaps more subtle differences in tone or note structure encode information about male attributes.

Although these results are consistent with the hypothesis that female songbirds could select better foragers as mates by assessing features of song, future work in wild populations is needed to confirm that male performance on artificial novel foraging tasks is correlated with foraging efficiency. While performance on another artificial measure of problem‐solving has been correlated with foraging efficiency in the wild (Cole et al., 2012), the specific lid‐flipping task that we used to measure novel foraging ability has not been. And while these results indicate that song may be a reliable signal of a fitness‐relevant cognitive task in the zebra finch, the songbird clade contains a huge variety of song types and singing behaviors (Catchpole & Slater, 2008). It is worth investigating whether females of other species also prefer songs from males who are better foragers, or whether certain species are able to encode this type of information better than others.

## CONFLICT OF INTEREST

We declare no conflicts of interest.

## AUTHOR CONTRIBUTION

**Clara Howell:** Conceptualization (equal); Investigation (equal); Writing‐original draft (lead); Writing‐review & editing (equal). **Rindy Anderson:** Conceptualization (equal); Writing‐review & editing (equal). **Elizabeth P. Derryberry:** Conceptualization (equal); Funding acquisition (lead); Investigation (equal); Writing‐review & editing (equal).

## ETHICAL APPROVAL

Ethical approval for this research was obtained from the Tulane University Animal Use Committee, Protocol 0427R.

## Supporting information

Appendix S1‐S4

## Data Availability

Available on Dryad (https://doi.org/10.5061/dryad.3tx95x6cp).
