# Supplementary material for: Female zebra finches prefer the songs of males who quickly solve a novel foraging task to the songs of males unable to solve the task
Source: Ecol Evol. 2020 Aug 20;10(18):10281–91. doi: 10.1002/ece3.6690 (PMC7520207; doi:10.1002/ece3.6690)
Supplement: Supplementary file 1 — Appendix S1‐S4 [file ECE3-10-10281-s001.docx]

*Female zebra finches prefer the songs of males who quickly solve a novel foraging task to the songs of males unable to solve the task*: Appendices

Contents:

I. Raw data for novel foraging task

II. Raw data for stimuli, including which males were selected as stimulus males and information on song complexity

III. Raw data for female preference assays, including conspecific/heterospecific trials and Solver/Non-Solver trials

IV. Mixed effects linear model results and model selection

Appendix S1: Raw data for male performance on novel foraging task

Appendix S2: Raw data for stimuli, including which males were selected as stimulus males and information on song


Appendix S3: Raw data for female preference trials

Conspecific vs. Heterospecific:

Solver vs. Non-Solver:

Appendix S4: Results from mixed-effects linear models:

We ran mixed effects linear models with stimulus set, female ID, and order of presentation as random effects. Including all random effects, the intercept was 0.56776 with a p-value of 2.37e-06. Including no random effects (the best model, according to AIC values), the intercept was still 0.5677 with a p-value of 2e-16. This is a smaller estimate of preference than we found with our Bayesian model, likely because this model placed equal weight on preferences with few overall hops and those with many overall hops and did not account for side bias. However, even using a frequentist approach, we see that females are significantly preferring Solver songs over the null.

| **Model** | **AIC value** |
| --- | --- |
| Preference~1 | -4.817 |
| Preference~1 + (1\|Stimulus) | -2.938 |
| Preference~1 + (1\|Female ID) | -2.817 |
| Preference~1 + (1\|Order) | -2.817 |
| Preference~1 + (1\|Stimulus) + (1\|Female ID) | -0.938 |
| Preference~1 + (1\|Stimulus) + (1\|Order) | -0.938 |
| Preference~1 + (1\|Female ID) + (1\|Order) | -0.817 |
| Preference~1 + (1\|Stimulus) + (1\|Female ID) + (1\|Order) | 1.062 |
